# Unclear Relationship: Prenatal but not Concurrent Bisphenol A Exposure Linked to Lower Weight and Less Fat

**DOI:** 10.1289/ehp.121-a135

**Published:** 2013-04-01

**Authors:** Kellyn S. Betts

**Affiliations:** For more than a dozen years Kellyn S. Betts has written about environmental contaminants, hazards, and technology for solving environmental problems for publications including *EHP* and *Environmental Science & Technology*.

The first prospective study to estimate effects of prenatal and early-life exposure to bisphenol A (BPA) on children’s body mass has found that girls who were exposed to the highest concentrations *in utero* had lower weight for their height and were less likely to be obese at age 9 than girls with the lowest exposures [*EHP* 121(4):514–520; http://dx.doi.org/1205548]. However, the same was not true of boys, and a cross-sectional analysis of both sexes at age 9 showed a positive association between current BPA urinary concentrations and obesity.

The children and their mothers were participants in the CHAMACOS (Center for the Assessment of Mothers and Children of Salinas) cohort study at the University of California, Berkeley. The mothers, most of them immigrant farmworkers, were pregnant in 1999 and 2000 when they were recruited by Berkeley researchers.

BPA is found in consumer products including polycarbonate water and food containers, epoxy-lined food cans, dental sealants, and thermal papers. Food is believed to be the major source of exposure.

BPA is one of the world’s most studied chemicals. Animal and *in vitro* studies show it is an endocrine disruptor that appears to interfere with estrogen, androgen, and thyroid hormone pathways. Many animal studies have linked perinatal exposure to environmentally relevant levels of BPA with increased body mass, suggesting it may be an obesogen. However, animals dosed with similar levels of the chemical in other studies have had decreased body mass or no change.

Further complicating the issue is the fact that, in many of these studies, the increase in body weight in association with prenatal BPA exposure wasn’t apparent until the animals reached sexual maturity. The new study’s association between prenatal exposure and decreased body mass was strongest for girls who had not yet reached puberty. Additional followup as the girls progress to sexual maturity should provide information to either support or refute the animal findings.

**Figure:**
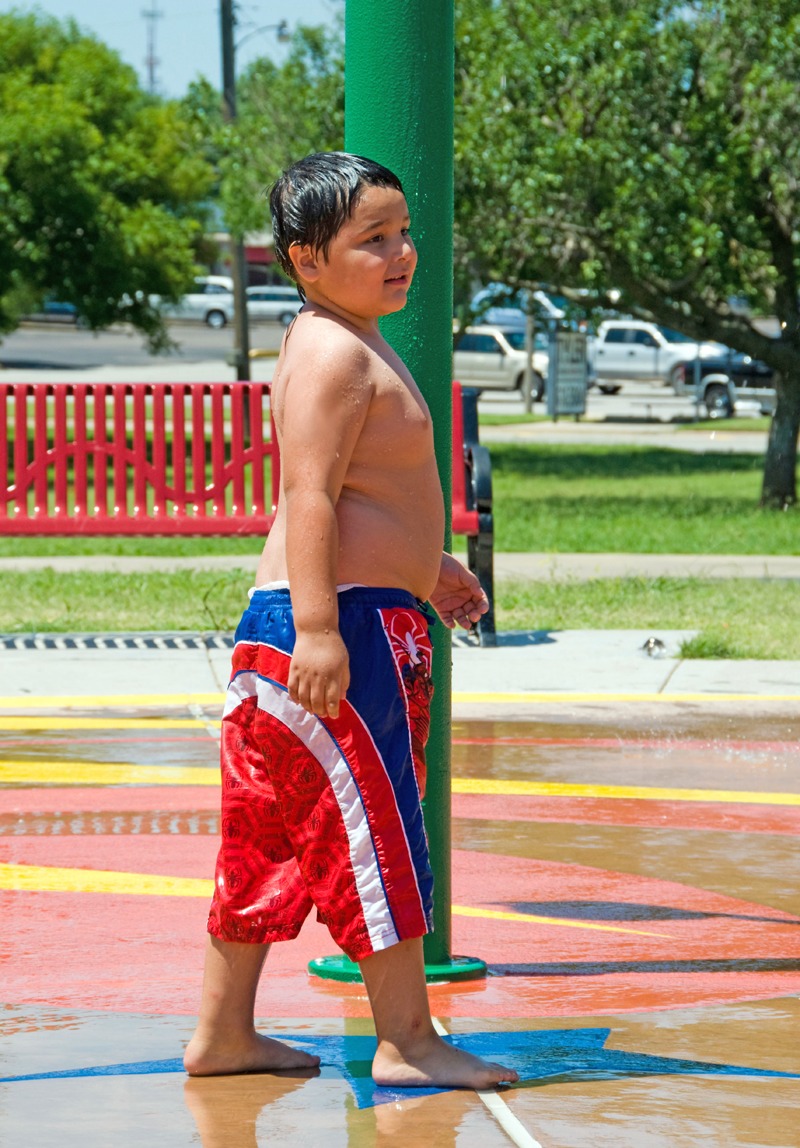
Cross-sectional studies have found an association between obesity and urinary levels of BPA, but it’s unclear whether BPA contributes to obesity or whether the diet of obese people results in greater BPA exposures. © AP Photo/Matt Rourke

The observed association between higher current concentrations of BPA in both male and female 9-year-olds with higher body fat and obesity levels is consistent with findings from other cross-sectional studies of adults and older children. The authors suggest that obese children may have higher urinary BPA levels because they eat more foods in plastic packaging or simply because they take in more calories—in other words, higher BPA levels could be a consequence of dietary habits that lead to obesity rather than a direct cause of obesity.

One limitation of this and other cross-sectional studies is that urinary BPA concentrations vary widely throughout the day, so a given sample is likely to reflect exposure only over the previous 4–6 hours. The Berkeley team plans to continue to follow the children to help clarify whether and how early BPA exposure is associated with body mass as they progress through puberty.

